# Atypical Presentation of Late-Onset Toxic Anterior Segment Syndrome Following Cataract Surgery: Challenges in Diagnosis and Management

**DOI:** 10.7759/cureus.79797

**Published:** 2025-02-27

**Authors:** Elie Breidy, Antoine El Sett, Jean Zalloum, Elias Chelala

**Affiliations:** 1 Surgery Department, Hôtel-Dieu de France, Beirut, LBN; 2 Faculty of Medicine, Saint Joseph University, Beirut, LBN; 3 Ophthalmology Department, Hôtel-Dieu de France, Beirut, LBN

**Keywords:** anterior chamber inflammation, endophthalmitis, intraocular pressure, toxic anterior segment syndrome, vitreous hemorrhage, vitritis

## Abstract

Toxic anterior segment syndrome (TASS) is a rare, non-infectious inflammatory response following anterior segment surgery that can be challenging to distinguish from infectious endophthalmitis, which requires different treatments. This case report describes a 72-year-old male with controlled hypertension, diabetes, and hyperlipidemia who developed an atypical presentation of TASS following uncomplicated cataract surgery. On postoperative day 7, the patient exhibited significant visual decline and anterior segment inflammation, raising concern for endophthalmitis. Diagnostic challenges included the absence of pain, delayed symptom onset, and vitritis. Treatment with intravitreal corticosteroids and antibiotics led to gradual visual recovery, with final visual acuity of 20/20 by day 30. No specific causative agent for TASS was identified, but the case highlights the importance of early recognition and management, as well as the potential contribution of surgical equipment issues and systemic risk factors in TASS development.

## Introduction

Toxic anterior segment syndrome (TASS) is a rare postoperative manifestation. It is known for its non-infectious inflammatory reaction following anterior segment surgery [1]. The incidence of TASS is estimated around 1/1000 after cataract surgery [2]. Although it is defined as an acute complication, late-onset TASS cases have been described in literature [1,3]. The major issue regarding this syndrome is the difficulty to differentiate it from infectious endophthalmitis, one of the most severe complications of ophthalmic surgery. In fact, each of these two entities requires a different treatment strategy and early diagnosis is mandatory to prevent poor visual outcomes [4].

Although TASS and endophthalmitis present some clinical key features that may be useful when it comes to differentiate one from the other, it seems like some cases can manifest in an unusual presentation, making the diagnosis more difficult [1,2]. This article aims to present a case of TASS with atypical presentation and the reflection made to confirm the diagnosis.

## Case presentation

A 72-year-old male patient was admitted to our department for cataract surgery in his right eye. The patient has a medical history of controlled hypertension, diabetes, dyslipidemia, and hyperuricemia, with a prior diagnosis of gout. Nearly 30 years ago, he was treated for high myopia with photorefractive keratectomy (PRK).

The patient’s left eye had been operated on four months earlier (for cataract surgery) without any complications. Postoperatively, his visual acuity (VA) in the left eye improved to 20/20. A MicroPure +10.5D intraocular lens (IOL) (PhysIOL S.A., Liege, Belgium) was implanted in that eye. Prior to surgery, the best-corrected VA (BCVA) in the right eye was 20/70. The patient underwent an uncomplicated phacoemulsification cataract surgery, during which a MicroPure 123 +11.0D hydrophobic IOL ((PhysIOL S.A.) was implanted.

The standard postoperative treatment consisted of two drops of prednisolone acetate (1%) and two drops of ciprofloxacin (0.3%) every two hours, along with a combination ointment of framycetin sulfate and dexamethasone sodium sulfate applied twice daily. A 24-hour postoperative check-up revealed no abnormalities, and the patient was advised to continue treatment and attend a follow-up visit one week later.

During the second check-up, the patient's VA in the right eye (OD) was measured at Counting Fingers at 15 cm. Intraocular pressure (IOP) in the right eye was 7 mmHg, and the pupil was normally reactive. The slit-lamp examination revealed fibrin debris and a 2-mm hypopyon. There was also limbus-to-limbus corneal edema, and a mild anterior chamber inflammatory reaction (graded 1+), along with the presence of a cyclitic membrane behind the posterior capsule. The fundus examination showed numerous inflammatory cells in the anterior vitreous body and traces of blood in the posterior vitreous, which made it impossible to evaluate the retina.

**Figure 1:**
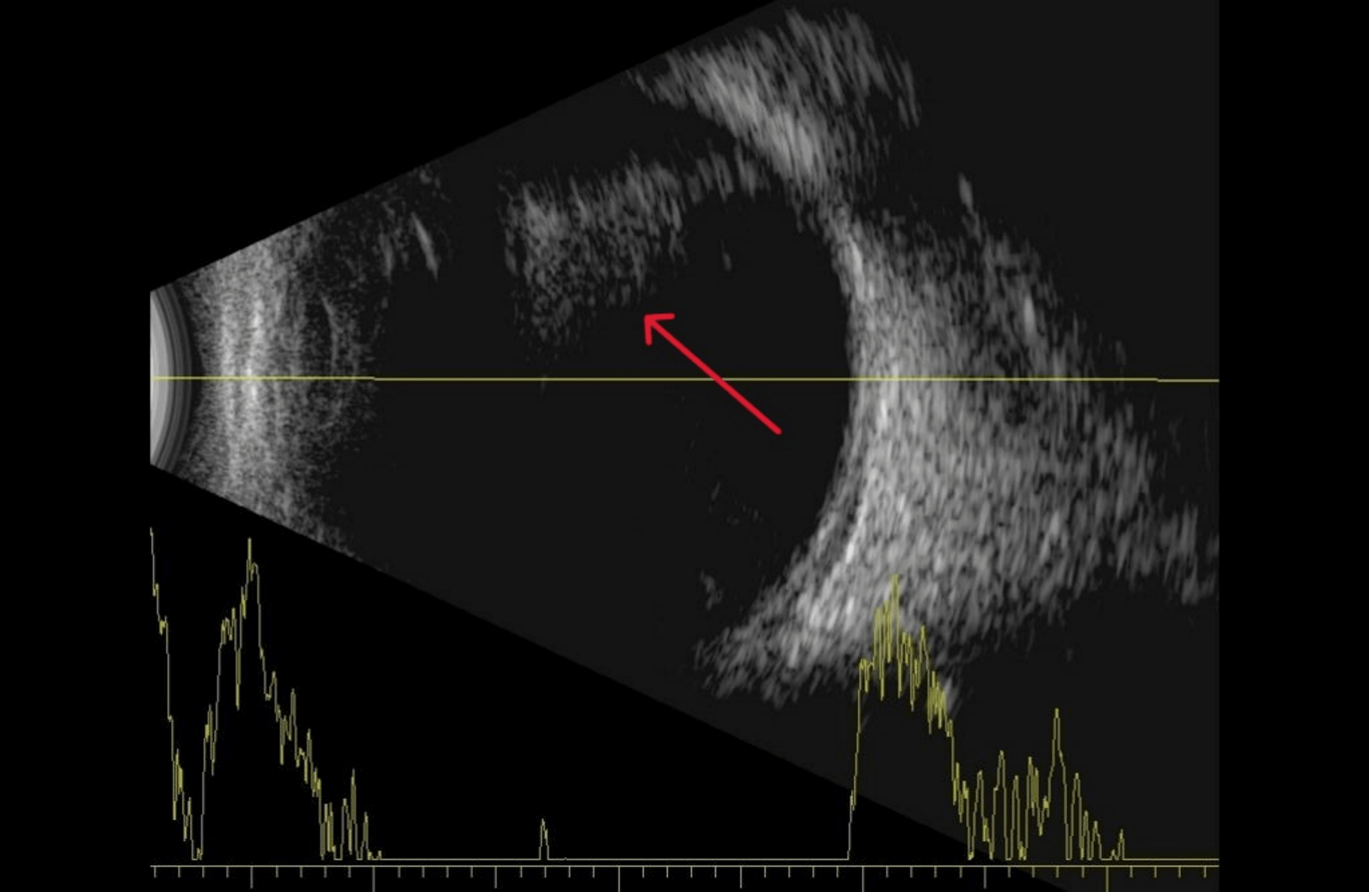
B-scan image showing opacities in the vitreous body.

A B-scan was performed, which confirmed our clinical suspicion of anterior vitritis and blood in front of the optic nerve (Figure 1). No retinal detachment was noted.

Upon further investigation, it was revealed that the patient had experienced mild head trauma shortly before his postoperative visit, with a rapid decline in VA following the incident. Importantly, the patient reported no pain at any point during his follow-up visits.

Given the clinical presentation, differentiating between late-onset TASS and infectious endophthalmitis proved challenging. Considering the severity of each potential diagnosis, a decision was made to administer an intravitreal injection of triamcinolone acetonide and gentamicin (80 mg/2 mL). Topical treatment with prednisolone acetate (1%) and moxifloxacin (5 mg/mL) was prescribed, with one drop every hour and every two hours, respectively. Cycloplegics were also administered. The patient was scheduled for a follow-up the next day.

On the following day, the VA in the right eye remained at Counting Fingers, and IOP was 9 mmHg, with no reported pain. A slight reduction in anterior chamber inflammation and anterior vitreous opacities was observed. The patient continued on the same regimen of antibiotics and corticosteroids.

After five days of treatment, the patient’s VA improved to 20/70, and IOP was measured at 10 mmHg. The slit-lamp examination revealed a calm anterior chamber, although the posterior capsule remained hazy. The fundus examination showed blood in the vitreous body, but there was a significant improvement in anterior vitritis. The retina was now visible and showed no signs of edema or abnormalities. The decision was made to maintain the current regimen of antibiotics and corticosteroids.

By day six, the patient’s VA had improved to 20/50, which led to the administration of a second intravitreal injection of triamcinolone acetonide and gentamicin (80 mg/2 mL). The following day, on day seven, a yttrium aluminum garnet (YAG) laser procedure was performed to address the persistent cyclitic membrane. Moving forward to day 10, a tapering of the corticosteroid regimen was initiated, and moxifloxacin was discontinued. As a result, by day 20, the patient’s VA had further improved to 20/25, with an IOP of 15 mmHg. The fundus examination revealed a clear vitreous body, free from inflammatory cells, prompting a modification of the topical treatment to include only prednisolone acetate, administered every three to four hours. By day 30, the patient’s VA had reached 20/20, and both the slit-lamp and fundus exams returned normal results. Consequently, the corticosteroid regimen was further tapered to one drop three times a day, with the medication being completely discontinued by day 40, marking the successful conclusion of the treatment.

## Discussion

When it comes to differentiating between TASS and infectious endophthalmitis, some characteristics may come in handy. First, the timing of occurrence can guide the diagnosis. TASS is generally known for its acute presentation (within 24 hours of the postoperative period) while infectious endophthalmitis occurs around three to seven days later [1]. However, many cases of delayed onset TASS have been reported [1,2,5], thus making the parameter of timing unreliable when it comes to differentiating TASS from infectious endophthalmitis. In our situation, the onset of TASS was not clearly determined, and could easily lead to confusion with endophthalmitis. It is certain that the inflammatory reaction was not visible after the first 24 hours, and that delay promoted doubt regarding an eventual infection.

The anterior vitritis aspect in addition to the blood of unknown origin we found on the funduscopy led us towards endophthalmitis. Nevertheless, the localization of inflammation in the anterior part of the vitreous and the capsular bag, in its majority, left us skeptical. Literature mentions that vitreous involvement tilts the balance in favor of infection; however, it does also suggest that “spillover” and vitritis, even if rare (less than 25% of cases), can occur especially in the anterior portion [5-8].

The fact that neither pain nor purulent discharge or any systemic sign was present reinforced our uncertainties. Certainly, one cannot rely on the absence of pain to eliminate the infection, but knowing that pain is found in 75% of endophthalmitis cases [9] helps us consider TASS as a possible diagnosis in such circumstances.

It is true that VA improvement was slow, but this can be attributed to the rate of resorption of the vitreous hemorrhage (1% per day) [10]. In fact, the inflammation of the anterior chamber and the anterior vitritis found initially were mostly resolved after five days of treatment. This strong responsiveness to the topical corticosteroids and the injection of triamcinolone, though insufficient as proof to justify the diagnosis, is characteristic of TASS [8].

Many studies discussed IOP variations in TASS. We found a clear divergence in the literature regarding this matter. The prevailing opinion states that a possible natural course of IOP in TASS starts with a decrease caused by the inflammation that results in reduced production of aqueous humor, only to be followed by an increase resulting from the blockage of the trabecular meshwork [1,11]. On the other hand, some studies mentioned that IOP was found significantly higher in the TASS group compared to normal patients IOP in the control group on the first postoperative day only, and still remained within the normal range [12]. Our follow-up revealed that the IOP was at its lowest when the diagnosis of TASS was made (a week after surgery). It strictly increased to reach its peak after 20 days of treatment and then stabilized at 15 mmHg. This evolution seems in accordance with what is found in most reviews. High IOP beyond normal ranges must have been avoided in this case due to early and aggressive treatment, avoiding permanent damage to the trabecular meshwork.

Identifying the etiology of TASS is very challenging. With a continuously expanding list of causal agents, a specific cause is not found in many cases [2]. We went through a thorough investigation to determine what could have possibly been implicated in the case we discussed. Preoperative viscoelastics, intracameral solutions, and balanced salt solutions (BSSs) were used correctly with our usual standard brands and dosages (0.5 mL of Shugarcain, 1 mg of diluted cefuroxime). No changes in our sterilization protocols or products were noted. However, a partially occluded irrigation/aspiration (I/A) tip was mentioned by the surgical team in the days following the surgery. It appears that the tip was occluded with a piece of residual lens that could not be removed by the sterilization process. Flushing under high pressure managed to take it out, thus showing the defect in the tip. Knowing that such elements were described as potential etiologies [13], we can assume that this defect in sterilization could possibly have been responsible for the TASS we experienced.

In addition, factors inherent to the patient can provide favorable conditions for the development of TASS. These factors such as type-2 diabetes, systemic hypertension, and hyperlipidemia, which are considered potential risk factors of TASS [14], were found in our patient’s medical records, in addition to some other inflammatory conditions that are not described as potential causal agents as of now, such as hyperuricemia and Gout disease.

## Conclusions

TASS is a rare but significant complication following cataract surgery, often presenting as an acute, non-infectious inflammatory response. The case described highlights the challenges in distinguishing TASS from infectious endophthalmitis, particularly when the presentation includes atypical features such as late-onset symptoms and the absence of pain or purulent discharge. Timely diagnosis is crucial, as early intervention with corticosteroids and appropriate therapy can lead to significant improvement in visual outcomes, as evidenced by the gradual recovery of the patient in this case. Although the exact cause of TASS in this patient remains unclear, potential contributing factors include a defect in surgical sterilization procedures and the patient’s pre-existing systemic conditions, such as diabetes and hypertension. This case emphasizes the importance of thorough clinical evaluation, careful monitoring, and swift treatment to mitigate the risk of poor visual outcomes associated with TASS.

## References

[1] Hernandez-Bogantes E, Navas A, Naranjo A, Amescua G, Graue-Hernandez EO, Flynn HW Jr, Ahmed I (2019). Toxic anterior segment syndrome: a review. Surv Ophthalmol.

[2] Verma L, Malik A, Maharana PK, Dada T, Sharma N (2024). Toxic anterior segment syndrome (TASS): a review and update. Indian J Ophthalmol.

[3] Li L, Zhou Q (2023). Late-onset toxic anterior segment syndrome after ICL implantation: two case reports. BMC Ophthalmol.

[4] Sengillo JD, Chen Y, Perez Garcia D, Schwartz SG, Grzybowski A, Flynn HW Jr (2020). Postoperative endophthalmitis and toxic anterior segment syndrome prophylaxis: 2020 update. Ann Transl Med.

[5] Suzuki T, Ohashi Y, Oshika T, Goto H, Hirakata A, Fukushita K, Miyata K (2015). Outbreak of late-onset toxic anterior segment syndrome after implantation of one-piece intraocular lenses. Am J Ophthalmol.

[6] Lee MH, Cugley D, Atik A, Ang GS (2017). Endophthalmitis or toxic anterior segment syndrome?. Clin Exp Optom.

[7] Park CY, Lee JK, Chuck RS (2018). Toxic anterior segment syndrome-an updated review. BMC Ophthalmol.

[8] Holland SP, Morck DW, Lee TL (2007). Update on toxic anterior segment syndrome. Curr Opin Ophthalmol.

[9] Ozcelik ND, Eltutar K, Bilgin B (2010). Toxic anterior segment syndrome after uncomplicated cataract surgery. Eur J Ophthalmol.

[10] Shaikh N, Srishti R, Khanum A (2023). Vitreous hemorrhage - causes, diagnosis, and management. Indian J Ophthalmol.

[11] Mamalis N, Edelhauser HF, Dawson DG, Chew J, LeBoyer RM, Werner L (2006). Toxic anterior segment syndrome. J Cataract Refract Surg.

[12] Shouchane-Blum K, Gershoni A, Mimouni M, Zahavi A, Segal O, Geffen N (2021). The association between toxic anterior segment syndrome and intraocular pressure. Graefes Arch Clin Exp Ophthalmol.

[13] Shouchane-Blum K, Dotan A, Bahar I (2019). The evolution of toxic anterior segment syndrome. Curr Opin Ophthalmol.

[14] Yazgan S, Celik U, Ayar O (2018). The role of patient's systemic characteristics and plateletcrit in developing toxic anterior segment syndrome after uneventful phaco surgery: a case-control study. Int Ophthalmol.

